# Assessing the Functional Properties of TiZr Nanotubular Structures for Biomedical Applications, through Nano-Scratch Tests and Adhesion Force Maps

**DOI:** 10.3390/molecules26040900

**Published:** 2021-02-09

**Authors:** Maria Vardaki, Aida Pantazi, Ioana Demetrescu, Marius Enachescu

**Affiliations:** 1 Center for Surface Science and Nanotechnology, University Politehnica of Bucharest, Splaiul Independentei 313, 060042 Bucharest, Romania; maria.vardaki@cssnt-upb.ro (M.V.); aida.pantazi@cssnt-upb.ro (A.P.); 2 S.C. NanoPRO START MC S.R.L., Mitropolit Antim Ivireanu Street 40, 110310 Pitesti, Romania; 3 General Chemistry Department, University POLITEHNICA of Bucharest, Gheorghe Polizu Street 1-7, 011061 Bucharest, Romania; 4 Academy of Romanian Scientists, Ilfov Street, 3, 50044 Bucharest, Romania

**Keywords:** TiZr nanotubes, surface roughness, force adhesion map, nano-scratch testing, hardness

## Abstract

In this work we present the results of a functional properties assessment via Atomic Force Microscopy (AFM)-based surface morphology, surface roughness, nano-scratch tests and adhesion force maps of TiZr-based nanotubular structures. The nanostructures have been electrochemically prepared in a glycerin + 15 vol.% H_2_O + 0.2 M NH4F electrolyte. The AFM topography images confirmed the successful preparation of the nanotubular coatings. The Root Mean Square (RMS) and average (Ra) roughness parameters increased after anodizing, while the mean adhesion force value decreased. The prepared nanocoatings exhibited a smaller mean scratch hardness value compared to the un-coated TiZr. However, the mean hardness (H) values of the coatings highlight their potential in having reliable mechanical resistances, which along with the significant increase of the surface roughness parameters, which could help in improving the osseointegration, and also with the important decrease of the mean adhesion force, which could lead to a reduction in bacterial adhesion, are providing the nanostructures with a great potential to be used as a better alternative for Ti implants in dentistry.

## 1. Introduction

Titanium (Ti) is a metallic material that, thanks to the native oxide layer formed on its surface, presents adequate resistance to corrosion in a variety of media, such as bioliquids, and good biocompatibility [1]. Titanium and its alloys–tissue interface reaction started to be intensively investigated [2] following Branemark’s [3] osseointegration concept, which led to its widespread use as a restorative biomaterial in the field of bioapplications [4,5].

The need for the continuous improvement of mechanical properties and the increase of antibacterial inhibition resulted in the use of new Ti alloys as well as new procedures, in order to meet the properties needed for improving the bio-performance of metallic biomaterials [6]. The alloying of Ti generally leads to an enhancement of its properties and alloying with zirconium (Zr) is, nowadays, considered a good choice for biomedical implants, especially in dentistry [7]. By alloying these two materials, their mechanical properties are enhanced [8], while their biocompatibility is boosted [9]. Zr, like Ti, possesses a native passive film formed on its surface that offers protection against corrosion, but its osseointegration is better than Ti [10].

The ratio between Ti and Zr of TiZr binary alloys can affect properties such as electrochemical stability and biocompatibility. Based on a series of investigations [11,12,13,14] that were performed, it was revealed that TiZr alloys with a percentage of 50% Zr [13] present polarization curves with a larger passive range, meaning that the resistance to localized corrosion is higher compared to other TiZr binary alloys, such as Zr5Ti and Zr25Ti alloys, which were susceptible to localized corrosion [11,12]. Moreover, the TiZr alloys with 50% Zr are considered to have superior mechanical properties [8,15] along with enhanced behavior in terms of biocompatibility [9,16].

It was reported that an increase of Zr content on the surface resulted in an increase of nanohardness [15]. Such a type of surface possesses a reduced modulus of elasticity, namely 77–98 GPa, which is significantly improved compared with Ti, which has a modulus of elasticity of 110 GPa. Nanoindentation experiments for TiZr coatings with the increase of the content of Zr from 11 to 22 wt.% exhibited an enhancement of the elastic strain to failure and plastic deformation resistance. It is worth mentioning that the TiZr alloys have an elastic modulus that can be adjusted to those of natural bones [17]. With such properties, TiZr alloys are the most promising biomaterials to be used in 3D printing of medical devices by using modern additive manufacturing technologies, and more knowledge will benefit applications. According to previous studies [18,19], it was revealed that the nanomechanical properties as well as the biocompatibility of the Ti50%Zr binary alloy can be affected by the condition of annealing treatments (in air, reduced in Ar/H2) that are followed after the anodizing procedure.

This manuscript focuses on the nanomechanical behavior of TiZr samples with a Ti to Zr mass ratio of 1:1 studied via Atomic Force Microscopy (AFM) investigation, after they have been subjected to a two-step electrochemical anodizing and various annealing post treatments. Due to very complex aspects regarding the correlation between morphological parameters (diameter, wall thickness) or thermal modification parameters (temperature, time, crystal structure) and the mechanical properties (roughness, hardness, etc.), the materials and the preparation and characterization procedures were chosen based on literature data [20,21,22,23] and our previous results [18,19,24]. The selection of the anodizing protocol and thermal modification parameters was made in order to obtain an improvement in performance, and in particular to improve biocompatibility and antibacterial effect [18,19]. The protocol of the two-step anodizing offers a higher level of organization of the nanostructures, due to the nanotexturing of the substrate that was achieved by ultrasonicating the sample after the first anodizing step [19]. As a result of comparing TiO_2_ nanotubes obtained in one vs. two anodizing steps it was established [25] that the essential difference between them is related to their architectures (length, diameter, porosity). According to references [18,19,20,21,22,23,24], the surfaces covered with TiO_2_ nanotubes with diameters in the range of 25–90 nm lead to better performances, such as corrosion biocompatibility and electrical conductivity, which are important for various applications as well as for other technological applications. The choice of the electrolyte is also highly important in the preparation of the nanostructures. The morphology of the obtained nanotubes can be affected by the viscosity of the electrolyte, since the mobility of the ions during the anodizing procedure is affected as well. Electrolytes of high viscosity, such as glycerol-based electrolytes, result in poor ion transmission and chemical dissolution, meaning that the ions move to a slower pace [26]. This ensures that the growth rate of the nanotubes is not high, and thus the final nanotubes will be smoother, more homogeneous and with a high organization level.

To the best of our knowledge, despite the relatively large number of papers about TiZr nanostructures, there are scarce data regarding their AFM morphology with nano-mechanical and adhesive aspects, and covering this gap is the aim of this investigation and its novelty.

## 2. Results and Discussion

### 2.1. Morphological Properties

**Figure 1:**
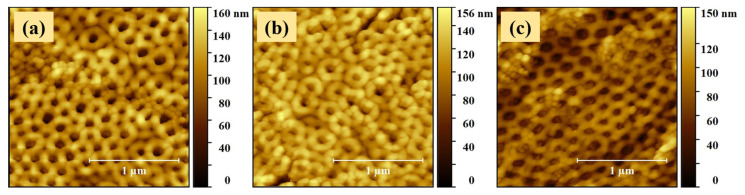
2D projection of the AFM topography images recorded for the (**a**) S1 (anodized), (**b**) S2 (anodized and air annealed) and (**c**) S3 (anodized, air annealed and reduced) samples.

There are various properties that have notable effects on the functionality of materials, and among these surface morphology is one of the most important [27]. A set of 2 × 2 µm^2^ representative AFM topography images of the prepared TiZr samples are presented in Figure 1. As can be observed, the TiZr based nanostructures exhibited a highly ordered spatial arrangement of the TiZr nanotubes, confirming the successful application of the two-step anodizing protocol. Tubular structures provide a big surface area that can affect the wettability as well as the adsorption of proteins and ions [28]. Moreover, the formation of a biofilm can be diminished thanks to nanotubular structures, and bacteria inhibition can be increased in the implant region leading to the assumption that it is less likely for the implant to be rejected [4].

The AFM images shown in Figure 1b,c do not reveal any significant influence of the air annealing and reduction post-treatments on the structural and/or topographical properties of the coatings. The nanotubes maintained their well-defined shapes, dimensions and spatial distributions.

The mean surface roughness parameters (Table 1) of the prepared TiZr coatings based on 2 × 2 µm^2^ AFM topography images were calculated for each sample by averaging the RMS and Ra values obtained from three different regions, including the ones illustrated in Figure 1. The similarity of the mean surface roughness parameters (RMS and Ra) exhibited by the anodized (30.3 ± 1.4 nm/24.1 ± 0.8 nm), anodized and air annealed (27.6 ± 1.4 nm/21.8 ± 1.6 nm) and anodized, air annealed and reduced (27.8 ± 2.3 nm/20.52 ± 1.9 nm) coatings underlines the comparable uniformity level of the surfaces, being described by similar grades of deviations from their mean height values. Yet, a slight decrease in the mean surface roughness parameters of about 8% could be observed for the samples subjected to the annealing treatment (S2 and S3).

**Figure 2:**
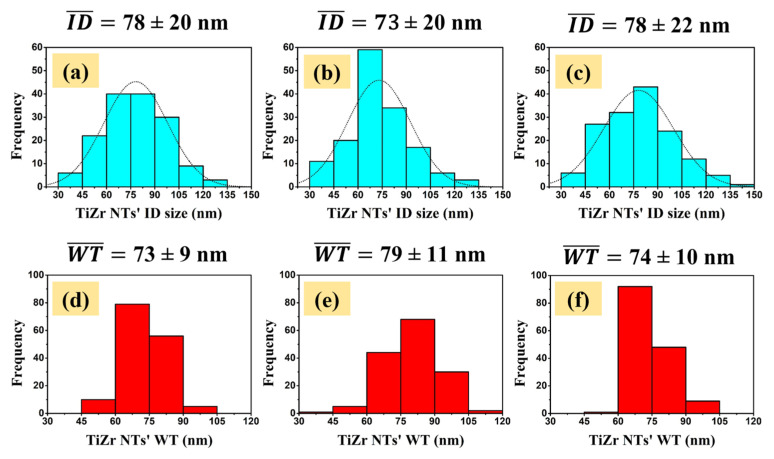
Distributions of the TiZr nanotubes’ ID sizes and WTs, respectively, for the (**a**,**d**) S1, (**b**,**e**) S2 and (**c**,**f**) S3 samples.

TiZr nanotubes’ inner diameter sizes (ID) and wall thicknesses (WT) distributions for all of the prepared coatings are presented in Figure 2. The nanotubes’ ID sizes and WTs were extracted from a series of there different 2 × 2 µm^2^ AFM topography images, by measuring around 150 individual nanotubes for each type of sample.

All three ID sizes histograms (Figure 2a–c) were best fitted with a normal function, revealing unimodal distributions. The S1 and S3 samples showed a higher statistical dispersion for the TiZr nanotubes’ inner diameter sizes, with dominant values within the 45–105 nm range, compared to the S2 sample that exhibited the highest number of nanotubes in a narrower range of sizes, namely 60–90 nm. The mean NTs ID sizes for the S1, S2 and S3 samples were found to be 78 ± 20 nm, 73 ± 20 nm and 78 ± 22 nm, respectively.

The d, e and f graphs, illustrated in Figure 2, present the TiZr NTs’ wall thickness distributions. As can be observed, sample S2 showed a slightly higher WT statistical dispersion, with most of the values in the range of 60–105 nm, compared to samples S1 and S3 that exhibited WTs ranging from 60 to 90 nm. The calculated mean WT value for the S1 (only anodized) sample was found to be 73 ± 9 nm, while the S2 sample revealed a mean WT of 79 ± 11 nm. This slight increase in the WT is, most likely, correlated with the decrease in the ID sizes of the nanotubes, and could be associated with an excessive ion diffusion at the walls, which induces further oxidation processes during annealing. These results confirm the data from our previous works on nanotubes, which showed that the annealing process induces modifications of surface features (i.e., length of nanotubes, diameter, wall thickness). The as-formed nanotubes are amorphous, but when annealed they become crystalline, passing to both anatase and rutile phases [24]. Literature data confirmed that the TiO_2_ nanotubes after the annealing process lead to a decrease of the diameter and an increase of the wall thickness. Annealing treatments affect the morphology of the nanotubes as a function of annealing temperature [24], this being drastic for samples annealed at higher temperatures such as 700 °C [22], where cracks appear in the oxide coating. Usually, the layer responsible for cracks and possible degradation of the nanotubes is a rutile phase [22].

### 2.2. Adhesive Properties

**Figure 3:**
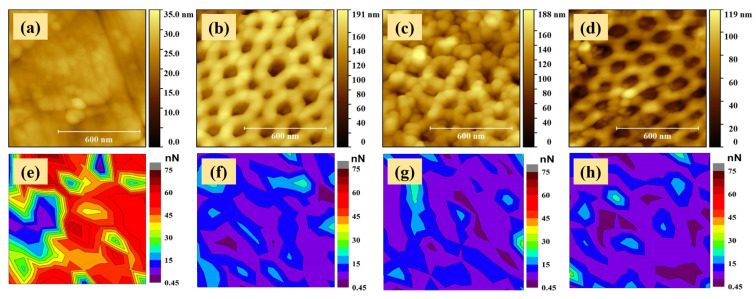
1 × 1 µm^2^ topography and adhesion force variation maps, respectively, for the (**a**,**b**) un-coated TiZr, (**b**,**f**) S1, (**c**,**g**) S2 and (**d**,**h**) S3 samples.

The adhesion force variation maps for the un-coated TiZr and nanotube-covered TiZr samples are illustrated in Figure 3e–h. The adhesion maps were recorded on the same sample location where the 1 × 1 µm^2^ topography images were acquired, by tracing a total number of 100 force-displacement curves. The adhesion force was calculated from each F-d curve, considering the pull-out region. 

**Figure 4:**
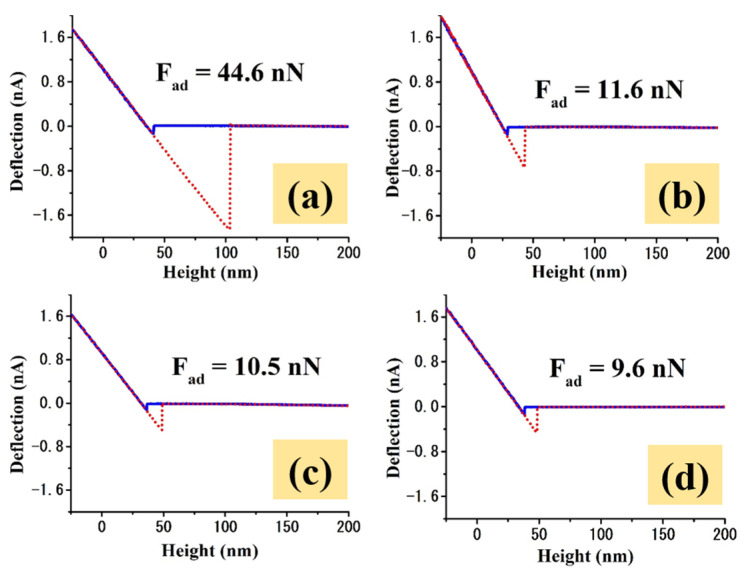
Representative F-d curves for the (**a**) un-coated TiZr, (**b**) S1, (**c**) S2 and (**d**) S3 samples.

A set of representative F-d curves for each investigated sample is presented in Figure 4.

In the imaged F-d measurements for the un-coated TiZr substrate (Figure 3e), the adhesion force values do not have a perfectly uniform distribution, also having some regional islands formed based on forces with comparable values. The adhesion force values exhibited by the un-coated TiZr range between 0.45 and 75.45 nN, with 40–60 nN as the most dominant force range, for which a mean adhesion force value of 51.7 ± 5.2 nN was obtained by averaging the forces that resulted from around 65 F-d measurements (Table 1).

Through the surface nanostructuring, as revealed by the adhesion map of sample S1 (Figure 3f), a significant influence on the adhesive properties is induced. S1 exhibited a considerably greater level of uniformity of adhesion forces’ distribution compared to the un-coated TiZr, exhibiting values ranging from 0.92 to 26 nN, with the most dominant force range being 5–20 nN; the un-coated TiZr revealed a significantly smaller mean adhesion force of 11.3 ± 3 nN, calculated by averaging around 80 data points. It is clear that the differences between the adhesion force distributions are the results of the surface morphological properties’ modifications. As a result of the anodization process that led to nanotubes formation, it is likely that the probe-surface inter-atomic/molecular attraction sites decreased due to the presence of the nano-holes, leading also to a decrease of the experienced mechanical adhesion. On the other hand, the decrease of the adhesion forces could also be related to the structural changes experienced by the materials, as the anodization process has oxidized the alloy. The adhesion force measured by AFM is the result of the interaction between the atoms from the tip and those from the sample, and therefore by changing the interaction material, the strength of the tip–sample interaction could also undergo changes.

The air annealing and reduction post-treatments had a noteworthy influence on the adhesive properties of the coatings, leading to a further (slight) decrease of the mean adhesion force values for samples S2 and S3, of 9.9 ± 2.2 nN and 9.3 ± 2 nN (Table 1), respectively, calculated by averaging around 80 measurements for each, based on their dominant force range, which was found to be 5–15 nN. The reductions in the adhesive properties of these surfaces could compromise the osseointegration and cell adhesion, but on the other hand, this decrease could also help in preventing bacterial adhesion, which could minimize the possibility (or magnitude) of the appearance of inflammatory processes at the tissue–implant interface, this being among the priorities in biomedical applications. Such an effect is very important in a time of very aggressive bacteria.

It is also worth mentioning that the TiZr samples’ surface roughness parameters have undergone an important increase after the anodizing protocol, from 8.7 ± 3 nm/6.6 ± 2 nm to 28.2 ± 0.7 nm/22.1 ± 0.8 nm (see Table 1), which in many cases has proven to be very beneficial for bio-applications by leading to an improved osseointegration and cell attachment [29]. The surface roughness increase together with the highly ordered nanotextured morphologies that, in fact, represent full “forests” of nanotubes, which could act as nano-connecters, make TiZr coatings potential candidates for becoming an alternative for Ti alloys.

### 2.3. Mechanical Properties

**Figure 5:**
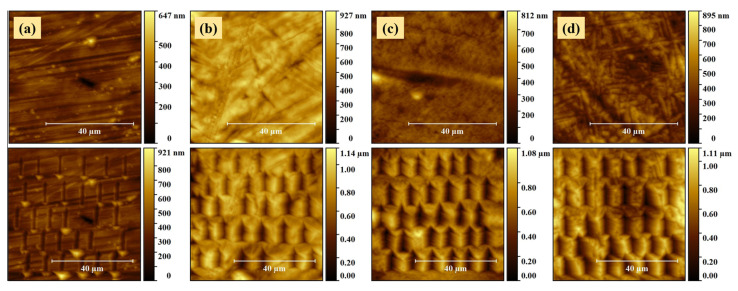
60 × 60 µm^2^ and 65 × 65 µm^2^ topography images of the (**a**) un-coated TiZr, (**b**) S1, (**c**) S2 and (**d**) S3 samples, recorded before (**top**) and after (**bottom**) performing the nano-scratch tests.

The mechanical properties of the TiZr samples were investigated using the nano-scratch testing method, in constant load mode. Figure 5 shows the topography images revealed by the un-coated and nanostructured TiZr samples, before (60 × 60 µm^2^) and after (65 × 65 µm^2^) tracing the nano-scratches.

The level of plastic deformation experienced by the samples is shown by the presence of the l-shaped imprints in the images recorded after completing the nano-scratch tests. The sizes of the imprints left by the scratches traced on the un-coated sample are smaller compared to the ones observed for the nanostructured coatings. The edges of the scratch imprints are surrounded by plastic deformation pile-ups, which were created as a results of the well-known volume conservation theory [30]. As expected and as can be seen in the topographical images, the material displaced by the indenter extruded sideways, forming dune-like shaped features along the scratches. This behavior is more evident in the case of nanostructured coatings.

**Figure 6:**
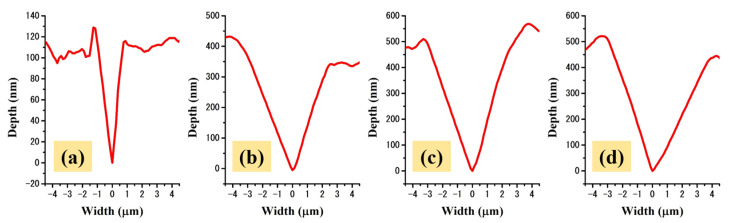
Representative scratch height vs. width profiles for the (**a**) un-coated, (**b**) S1, (**c**) S2 and (**d**) S3 TiZr samples.

Figure 6 displays the graphical representations of the scratch profiles for all of the analyzed TiZr based samples. As can be seen, the width of the scratch traced on the un-coated sample is significantly smaller compared to those exhibited by the coatings, a behavior that attributes a higher mechanical resistance to the TiZr substrate.

**Figure 7:**
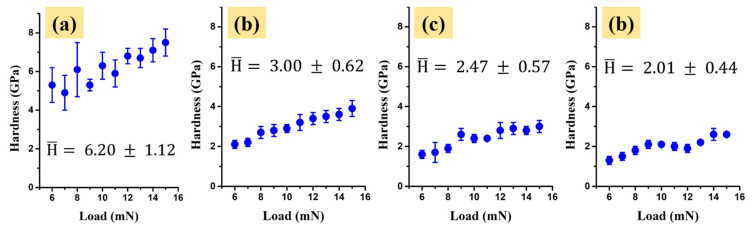
The mean hardness values calculated for the (**a**) un-coated, (**b**) S1, (**c**) S2 and (**d**) S3 TiZr samples.

In Figure 7 the hardness (H) parameters of the TiZr-based samples plotted as a function of the scratch’s applied force are illustrated. The H values shown in Figure 7 are the mean values of at least 10 measurements extracted as profiles from the three traced scratches using the same load, along with their corresponding deviation bars.

As indicated by the topography images through the scratch imprint widths, the un-coated TiZr sample revealed the highest mean hardness (H) parameter value of 6.20 ± 1.12 GPa. The S1 anodized coating showed a smaller mechanical resistance, highlighted by its mean hardness values of 3.00 ± 0.62 GPa. This behavior is expected, as the anodization process led to the formation of the nanotubes, which, most likely, facilitate the penetration of the indenter. The S2 and S3 samples showed slightly smaller mean H values of 2.47 ± 0.57 and 2.01 ± 0.44 GPa, respectively, compared to S1. This H decrease could, most likely, be associated with the applied air annealing and reduction post-treatments. A correlation between the structure of the nanotubes’ layer and the hardness can be observed, given that the unheated sample with amorphous nanotubes has a high hardness and after annealing this value decreases. This fact has been highlighted previously, simultaneously with the appearance of crystallinity [18]. In general, the as-formed TiO_2_ nanotubes are amorphous, but in the case of two-step anodizing, the final coatings of the nanotubes have a hexagonal crystalline phase before annealing. After annealing, a change in the crystal system occurs, passing from hexagonal to orthorhombic, causing changes in the mechanical properties as well. The conversion of the crystalline phase leads to a decrease in hardness due to the increased volume and d spacing parameters of the new structure, which could be possibly translated into a decrease in material density [19]. Reference [22] showed that the annealing temperature and the conversion of crystalline phases of the TiO_2_ nanotubes affect the mechanical and tribological properties of the oxide layer. A comparison of TiO_2_ nanotubes with an anatase crystal structure revealed that their mechanical properties, such as hardness and an elastic modulus, are lower than those exhibited by the Ti substrate, which presents abrasive and adhesive wear.

However, even though the samples showed a decrease in the mechanical properties, they still have great potential for biomedical applications due to their nanostructured morphologies that led to an increase of the surface roughness after anodizing, which could considerably enhance the osseointegration of the materials. Moreover, the adhesive properties of the coatings have been significantly reduced after anodizing, air annealing and reduction post-treatments, this result also being very promising as it could lead to an increase of the bacterial adhesion inhibition index.

## 3. Materials and Methods

### 3.1. Preparation of TiZr Nanostructures

The TiZr alloys, with 50% Zr and 50% Ti, were purchased from ATI Wah Chang Co., (American manufacturing company in the metal and alloy industry based in Albany, Oregon, USA). Before modifying the surfaces by applying the anodizing protocol, the TiZr substrates were mechanically polished with a premium SiC abrasive paper (1200 grit), after which they were cleaned in an ultrasonic bath with acetone (5 min), ethanol (5 min) and distilled water (5 min), finally being dried under a N_2_ stream.

The TiZr nanostructures were obtained using a two-step anodizing protocol in a mixture of glycerin + 15 vol.% H_2_O + 0.2 M NH4F, involving a typical two-electrodes electrochemical cell with a Pt counter electrode and the TiZr substrate used as a working electrode. Both anodizing steps were performed using the same electrolyte, but with different potentials and periods of time, namely 55 V for 4 h and 75 V for 1 h. In between the two anodizing steps, the samples were sonicated in water in order to remove the oxide layer formed on the surfaces in the first step. The resultant TiZr nanocoatings were cleaned in both ethanol and distilled water, and dried under a N_2_ stream. Subsequently, the samples were subjected to other post-treatments as follows: annealing using a tube furnace at 450 °C in air for 1 h, and reduction in Ar/H2 10% at 600 °C for 1 h (Table 2).

### 3.2. Characterization Methods

The morphological properties (topography and surface roughness parameters) of the samples were investigated in semicontact mode, at two different scan sizes (2 × 2 µm and 1 × 1 µm) using a high-resolution multimode Atomic Force Microscopy (AFM) system (NT-MDT Spectrum Instruments, Zelenograd, Russia), in ambient conditions, at a temperature (T) of 25 ± 0.5 °C and a relative humidity (RH) of 45 ± 3%, using cone-shaped tips made of monocrystalline silicon with an approximate radius of curvature of 10 nm, mounted on cantilevers with a stiffness of about 17 N/m.

The Root Mean Square (RMS) and the Average (Ra) roughness parameters were calculated based on the recorded topography images via an image processing software, using the following equations:

$$\begin{array}{c} RMS\;=\;\sum_{i=1}^{N}{\left[\frac{{\left(h_{i}\;-\;\bar{h}\right)}^{2}}{N}\right]}^{1/2};\\ R_{a}=\;\frac{1}{N}\;\sum_{i=1}^{N}\left|h_{i}-\bar{h}\right|, \end{array}$$

where *h_i_*, represents the height value at each data point, *h* represents the profile mean value of the surface, and *N* represents the number of data points in the analyzed profile.

The adhesive properties of the TiZr samples were studied through the AFM spectroscopy mode by placing a total number of 100 force-displacement (F-d) curves in a grid of 10 × 10 points, using silicon probes with a stiffness of about 0.65 N/m. The F-d curves were traced on the same sample locations where the 1 × 1 µm topography images have been recorded. The adhesion forces of the TiZr samples were calculated from the pull-out region of the force-displacement curves using Hook’s law:

F=k·Δz,

where *k* is the cantilever stiffness.

The mechanical properties, namely the hardness parameters (H), of the TiZr samples were evaluated through the micro-scratch testing method, performed using a three-sided pyramidal Berkovich diamond indenter with an apex curvature radius of about 70 nm. For each sample around 30 scratches were placed vertically on a 60 × 60 µm^2^ surface area by varying the applied loads from 6 to 15 mN. Each micro-scratch was traced by indenting the sample at a pre-defined load, followed by a top–down vertical sliding in constant load mode, along the surface up to the limit point. The resulting scratches were then attentively analyzed by extracting their average widths and the H parameters were calculated using the dedicated NanoScan Viewer processing software, using the following formula:

$$H=\frac{kP}{w^{2}},$$

where *P* is the applied load, *k* is a constant and *w* is the width.

## 4. Conclusions

In this work the functional properties of TiZr nanotubular coatings for biomedical applications have been assessed through AFM-based morphology, surface roughness, nano-scratch tests and adhesion force maps. Nanostructured TiZr coatings have been prepared in a mixture of glycerin + 15 vol.% H_2_O + 0.2 M NH4F, using a two-step anodizing procedure in a typical two-electrodes electrochemical cell.

The AFM topography images confirmed the formation of highly ordered nanotubular features on the prepared samples, with a mean inner nanotubes’ diameter (ID) size and wall thickness of 78 ± 20 nm and 73 ± 9 nm, respectively. The applied air annealing post-treatment slightly influenced surfaces’ morphological properties, leading to a small increase of NTs’ ID with the decrease of the their WT, being associated with an excessive ion diffusion at the walls, which induces further oxidation processes during annealing.

The surface roughness parameters significantly increased after the anodizing process compared to the un-coated TiZr substrate, whereas the mean adhesion force suffered an important decrease. The post-treatments also led to a further slight decrease of the adhesive properties. The reductions in the adhesive properties of these surfaces could help in preventing bacterial adhesion, with such an effect being very important in a time of highly aggressive bacteria.

On the other hand, the nanostructured coatings revealed smaller mean scratch hardness values compared to the un-coated sample. Yet, the scratch hardness values revealed by the coatings are still noteworthy and, most likely, capable of offering reliable mechanical resistance for various biomedical applications. Moreover, these functional properties come with a surface roughness increase that is known as beneficial for osseointegration, and with a decrease of the mean adhesion force that could minimize the possibility of inflammatory processes’ appearance at the tissue–implant interface, this being among the priorities in biomedical applications. Of course, further investigations are needed to better understand the behavior of these nanotubular coatings and to unlock the keys that will allow easy tuning of their properties.

## Figures and Tables

**Table 1:** Root Mean Square (RMS), Average (Ra) surface roughness parameters and the mean adhesion force values of TiZr samples.

Sample	Scan Size				Dominant F_ad_ Range [nN]	$\bar{F_{ad}}$ [nN]
	2 × 2 µm^2^		1 × 1 µm^2^			
	RMS (nm)	Ra (nm)	RMS (nm)	Ra (nm)		
TiZr un-coated	-	-	8.7 ± 3	6.6 ± 2	40–60	51.7 ± 5.2
S1	30.3 ± 1.4	24.1 ± 0.8	28.2 ± 0.7	22.1 ± 0.8	5–20	11.3 ± 3
S2	27.6 ± 1.4	21.8 ± 1.6	20.8 ± 3.5	16.5 ± 3	5–15	9.9 ± 2.2
S3	27.8 ± 2.3	20.52 ± 1.9	22.1 ± 3.7	16.6 ± 2.9	5–15	9.3 ± 2

**Table 2:** TiZr samples’ elaboration protocols, post-treatments and coding.

Sample Code	Elaboration Protocol	Post-Treatment
X	un-coated	–
S1	2-step anodizing	–
S2	2-step anodizing	air annealing
S3	2-step anodizing	air annealing and reduction

## Data Availability

The data presented in this study are available in this article.

## References

[1] Alves V.A., Reis R.Q., Santos I.C.B., Souza D.G., de Goncalves T.F., Pereira-da-Silva M.A., Rossi A., da Silva L.A. (2009). In situ impedance spectroscopy study of the electrochemical corrosion of Ti and Ti-6Al-4V in simulated body fluid at 25 degrees C and 37 degrees C. Corros. Sci..

[2] Takao H. (2019). Titanium–Tissue interface reaction and its control with surface treatment. Front. Bioeng. Biotechnol..

[3] Brånemark P.I. (1983). Osseointegration and its experimental background. J. Prosthet. Dent..

[4] Stoian A.B., Vardaki M., Ionita D., Enachescu M., Brancoveaun O., Demetrescu I. (2018). Nanopores and nanotubes ceramic oxides elaborated on titanium alloy with zirconium by changing anodization potentials. Ceram. Int..

[5] Popa M.V., Demetrescu I., Iordachescu D., Cimpean A., Vasilescu E., Drob P. (2007). The relation between electrochemical tests and in vitro evaluation of titanium alloy biocompatibility. Mater. Corros..

[6] Zhang L.C., Chen L.Y. (2019). A review on biomedical titanium alloys: Recent progress and prospect. Adv. Eng. Mater..

[7] Ionita D., Pirvu C., Stoian A.B., Demetrescu I. (2020). The trends of TiZr alloy research as a viable alternative for Ti and Ti16Zr Roxolid dental implants. Coatings.

[8] Cui W., Liu Y. (2019). Fatigue behavior of Ti50Zr alloy for dental implant application. J. Alloys Compd..

[9] Ion R., Mazare A., Dumitriu C., Pirvu C., Schmuki P., Cimpean A. (2018). Nanochannelar topography positively modulates osteoblast differentiation and inhibits osteoclastogenesis. Coatings.

[10] Trinca L.C., Mareci D., Souto D., Lozano-Gorrín A.D., Izquierdo J., Burtan L., Motrescu I., Vulpe V., Pavel G., Strungaru S. (2019). Osseointegration evaluation of ZrTi alloys with hydroxyapatite-zirconia-silver layer in pig’s tibiae. Appl. Surf. Sci..

[11] Mareci D., Bolat G., Chelariu R., Sutiman D., Munteanu C. (2013). The estimation of corrosion behaviour of ZrTi binary alloys for dental applications using electrochemical techniques. Mater. Chem. Phys..

[12] Akimoto T., Ueno T., Tsutsumi Y., Doi H., Hanawa T., Wakabayashi N. (2018). Evaluation of corrosion resistance of implant-use Ti-Zr binary alloys with a range of compositions. J. Biomed. Mater. Res. B.

[13] Moreno J.M.C., Popa M., Ivanescu S., Vasilescu C., Drob S.I., Neacsu E.I., Popa M.V. (2014). Microstructure, mechanical properties, and corrosion resistance of Ti-20Zr alloy in undoped and NaF doped artificial saliva. Met. Mater. Int..

[14] Chelariu R., Mareci D., Munteanu C. (2012). Preliminary electrochemical testing of some Zr–Ti alloys in 0.9% NaCl solution. Mater. Corros..

[15] Ivanova A.A., Surmeneva M.A., Shugurov V.V., Koval N.N., Shulepov I.A., Surmenev R.A. (2018). Physico-mechanical properties of Ti-Zr coatings fabricated via ion-assisted arc-plasma deposition. Vacuum.

[16] Ion R., Stoian A.B., Dumitriu C., Grigorescu S., Mazare A., Cimpean A., Demetrescu I., Schmuki P. (2015). Nanochannels formed on TiZr alloy improve biological response. Acta Biomater..

[17] Wen C.E., Yamada Y., Hodgson P.D. (2006). Fabrication of novel TiZr alloy foams for biomedical applications. Mater. Sci. Eng. C.

[18] Vardaki M., Mohajernia S., Pantazi A., Nica I.C., Enachescu M., Mazare A., Demetrescu I., Schmuki P. (2019). Post treatments effect on TiZr nanostructures fabricated via anodizing. J. Mater. Res. Technol..

[19] Pantazi A., Vardaki M., Mihai G., Ionita D., Stoian A.B., Enachescu M., Demetrescu I. (2020). Understanding surface and interface properties of modified Ti50Zr with nanotubes. Appl. Surf. Sci..

[20] Zalnezhad E., Baradaran S., Bushroa A.R., Sarhan A.A.D. (2014). Mechanical property enhancement of Ti-6Al-4V by multilayer thin solid film Ti/TiO_2_ nanotubular array coating for biomedical application. Metall. Mater. Trans. A.

[21] Arkusz K., Paradowska E., Nycz M., Krasicka-Cydzik E. (2018). Influence of thermal modification and morphology of TiO_2_ nanotubes on their electrochemical properties for biosensors applications. J. Nanosci. Nanotechnol..

[22] Fontes A.C.A., Sopchenski L., Laurindo C.A., Torres R.D., Popat K.C., Soares P. (2020). Annealing temperature effect on tribocorrosion and biocompatibility properties of TiO_2_ nanotubes. J. Bio Tribo Corros..

[23] Nycz M., Paradowska E., Arkusz K., Pijanowska D.G. (2020). Influence of geometry and annealing temperature in argon atmosphere of TiO2 nanotubes on their electrochemical properties. Acta Bioeng. Biomech..

[24] Mazare A., Ionita D., Totea G., Demetrescu I. (2014). Calcination condition effect on microstructure, electrochemical and hemolytic behavior of amorphous nanotubes on Ti6Al7Nb alloy. Surf. Coat. Technol..

[25] Yan S., Chen Y., Wang Z., Han A., Shan Z., Yang X., Zhu X. (2017). Essential distinction between one-step anodizing and two-step anodizing of Ti. Mater. Res. Bull..

[26] Ying Chin L., Zainal Z., Khusaimi Z., Ismail S.S. (2016). Electrochemical synthesis of ordered titania nanotubes in mixture of ethylene glycol and glycerol electrolyte. Malays. J. Anal. Sci..

[27] Gerber C., Lang H.P. (2006). How the doors to the nanoworld were opened. Nat. Nanotechnol..

[28] Beltrán-Partida E., Valdez-Salas B., Escamilla A., Curiel M., Valdez-Salas E., Nedev N., Bastidas J.M. (2016). Disinfection of titanium dioxide nanotubes using super-oxidized water decrease bacterial viability without disrupting osteoblast behavior. Mater. Sci. Eng. C.

[29] Paterlini T.T., Nogueira L.F.B., Tovani C.B., Cruz M.A.E., Derradi R., Ramos A.P. (2017). The role played by modified bioinspired surfaces in interfacial properties of biomaterials. Biophys. Rev..

[30] Gaillard Y., Tromas C., Woirgard J. (2006). Study of the dislocation structure involved in a nanoindentation test by atomic force microscopy and controlled chemical etching. Acta Mater..

